# Supplementary material for: Identification of bacterial signals that modulate enteric sensory neurons to influence behavior in C. elegans
Source: bioRxiv. 2025 Sep 3:2025.09.03.674032. Preprint. [Version 1] doi: 10.1101/2025.09.03.674032 (PMC12425002; doi:10.1101/2025.09.03.674032)
Supplement: 1 [file NIHPP2025.09.03.674032v1-supplement-1.pdf]

bioRxiv preprint doi: https://doi.org/10.1101/2025.09.03.674032; this version posted September 3, 2025. The copyright holder for this preprint (which was not certified by peer review) is the author/funder, who has granted bioRxiv a license to display the preprint in perpetuity. It is made available under aCC-BY 4.0 International license.

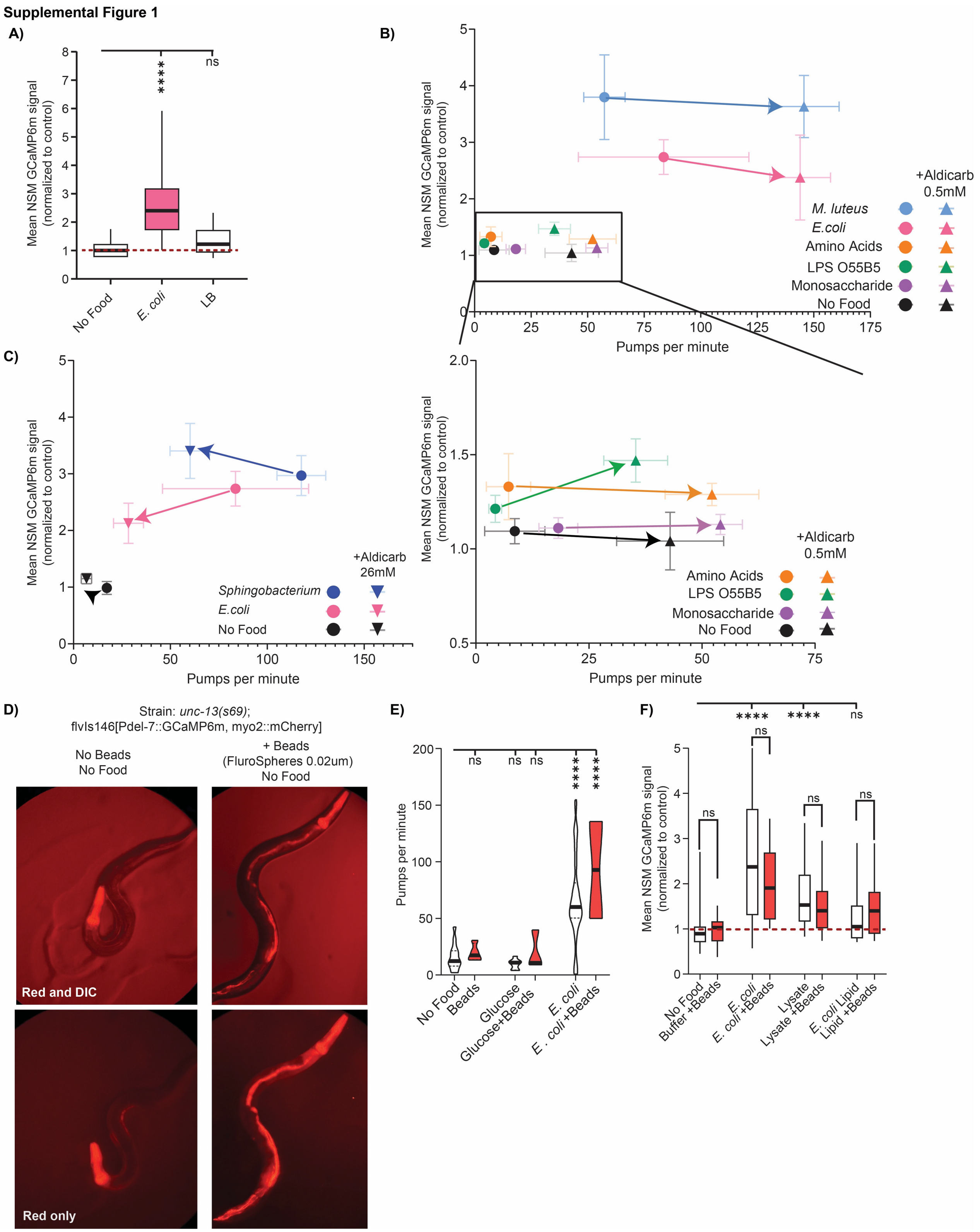

Supplementary Figure 1

(A) Mean GCaMP signal from NSM in unc-13(s69) animals, including Luria-Bertani (LB) broth negative control, shown as in Fig. 1C. ****p<0.0001 by Bonferroni-corrected Mann-Whitney test. n =12-45 animals.

bioRxiv preprint doi: https://doi.org/10.1101/2025.09.03.674032; this version posted September 3, 2025. The copyright holder for this preprint (which was not certified by peer review) is the author/funder, who has granted bioRxiv a license to display the preprint in perpetuity. It is made available under aCC-BY 4.0 International license.

(B) Scatter plot showing relationship between pumping and NSM activity (see main text for rationale). Circle dots represent mean NSM GCaMP signals from *unc-13(s69)* animals under various conditions without aldicarb. Triangles represent mean NSM GCaMP signals from animals treated with 0.5 mM aldicarb to increase pumping. Arrows connect data from the same experimental conditions where the only difference is aldicarb addition. Top: zoomed out plot. Bottom: a zoomed-in view of the inset box for clarity. Error bars are SEM. n=7-20 animals.

(C) Scatter plot showing relationship between pumping and NSM activity (see main text for rationale). Circle dots represent mean NSM GCaMP signals from *unc-13(s69)* animals under various conditions without aldicarb. Inverted triangles represent NSM signals from animals treated with 26 mM Aldicarb to reduce pumping. Arrows connect data from the same experimental conditions where the only difference is aldicarb addition. Error bars are SEM. n=11-22 animals.

(D) Representative images of *unc-13(s69)*; *flvIs146*[*del-7::GCaMP6m, myo2::mCherry*] animals with and without addition of FluroSphere 0.02um red fluorescent beads to the agar surface. These images were collected in the absence of food, in order to test whether the low basal pumping rate in the absence of food is sufficient for animals to ingest the contents on the agar surface. The +Beads images (right) show that red beads are clearly visible in the intestinal tract of the animal even under these no food conditions, indicating that substantial ingestion occurs even with a fairly low pumping rate. Note that the red color in the pharynx is the fluorescent co-injection marker also present in the absence of beads (left). Top images include the red and DIC channel to show the body outline of the animals; bottom images just show red fluorescence. Animals were permitted to ingest beads for 10 minutes prior to imaging (see Methods)

(E) Pharyngeal pumping rates of *unc-13(s69)* animals, exposed to the indicated substances for 15 minutes. n = 8-45 animals.

(F) Mean GCaMP signal from NSM in *unc-13(s69)* animals, for the indicated experimental conditions, which include the addition of the beads shown in (D). n =8-30 animals.

bioRxiv preprint doi: https://doi.org/10.1101/2025.09.03.674032; this version posted September 3, 2025. The copyright holder for this preprint (which was not certified by peer review) is the author/funder, who has granted bioRxiv a license to display the preprint in perpetuity. It is made available under aCC-BY 4.0 International license.

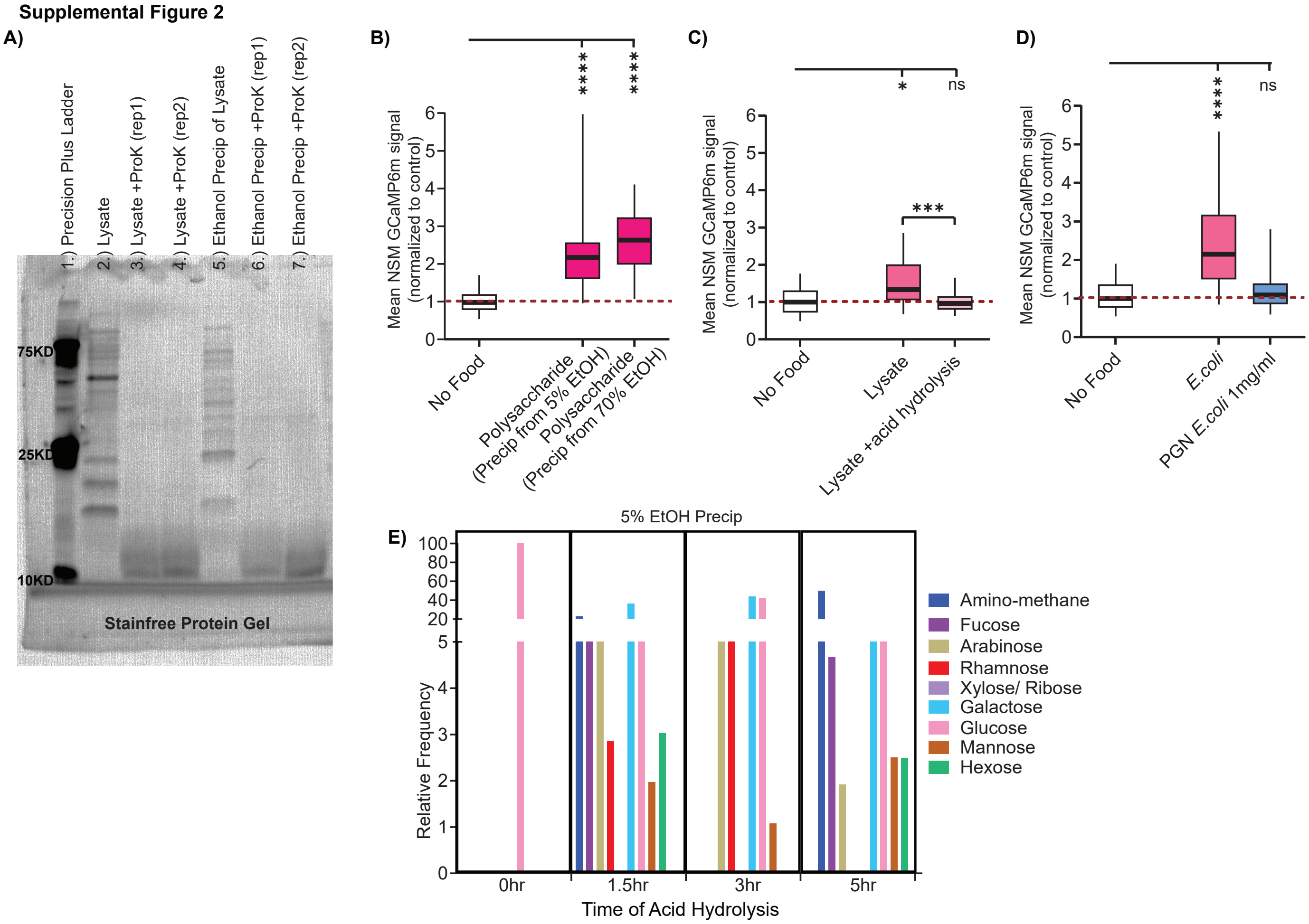

Supplementary Figure 2

(A) Stainfree protein gel showing proteinase K digestion of clarified lysate (compare lane 2 with lanes 3 and 4) and ethanol precipitated sample subjected to RNase, DNase, and Proteinase K treatment (compare lane 5 with lanes 6 and 7).

(B) Mean GCaMP signal from NSM in *unc-13(s69)* animals on indicated conditions, shown as in Fig. 1C. ****p<0.0001 by Mann-Whitney test. n = 35-48 animals.

(C) Mean GCaMP signal from NSM in *unc-13(s69)* animals on indicated conditions, shown as in Fig. 1C. ***p<0.001, *p<0.01 by Mann-Whitney test. n = 38-45 animals.

(D) Mean GCaMP signal from NSM in *unc-13(s69)* animals on indicated conditions, shown as in Fig. 1C. ****p<0.0001 by Mann-Whitney test. n = 42-120 animals.

(E) GC-MS carbohydrate profiling of a purified bacterial polysaccharide sample, shown as shown in Fig. 2D. Whereas the dataset in Fig. 2D was from a 70% ethanol precipitation, this sample was precipitated with 5% ethanol; the results are qualitatively similar across both samples.

bioRxiv preprint doi: https://doi.org/10.1101/2025.09.03.674032; this version posted September 3, 2025. The copyright holder for this preprint (which was not certified by peer review) is the author/funder, who has granted bioRxiv a license to display the preprint in perpetuity. It is made available under aCC-BY 4.0 International license.

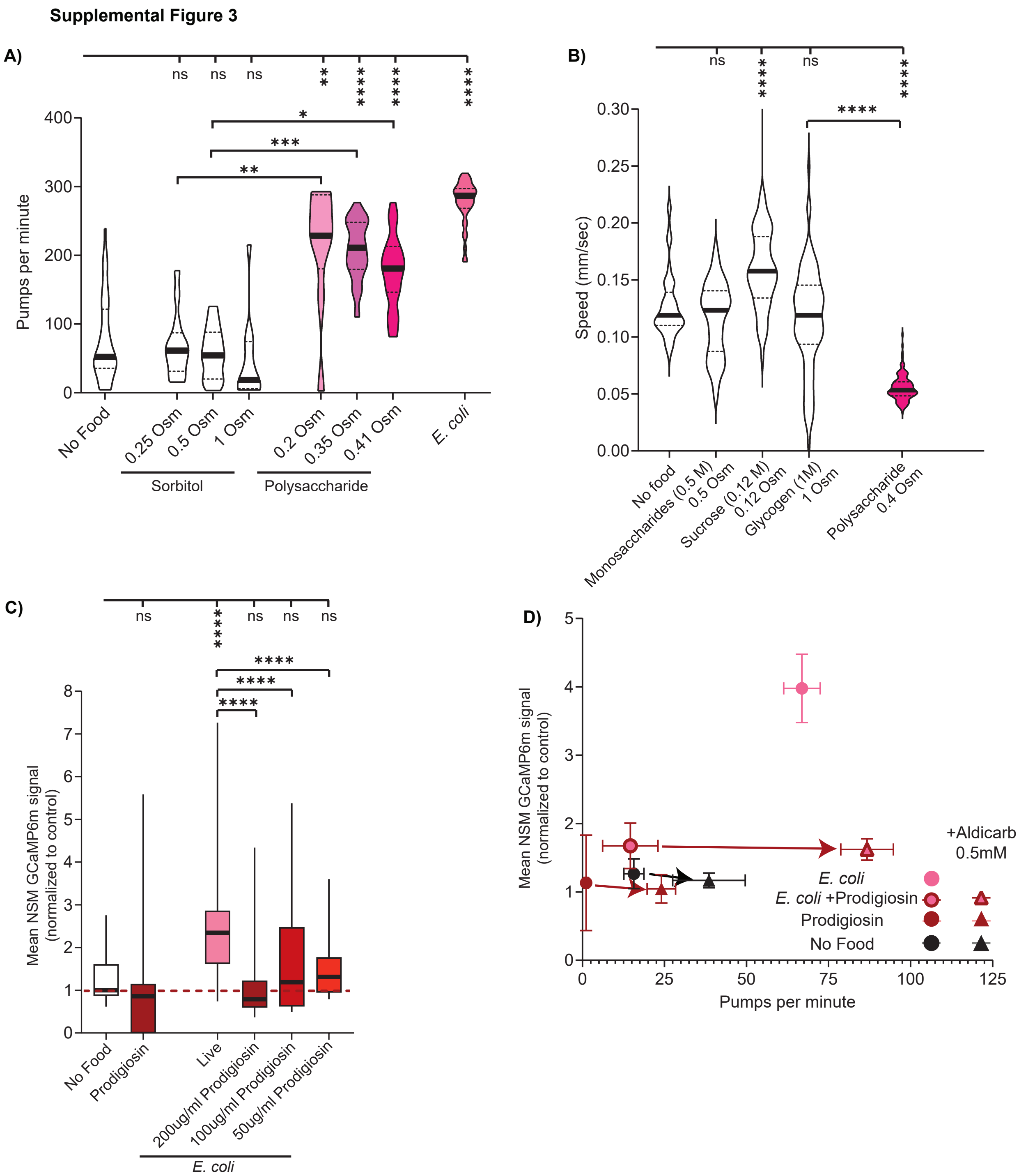

Supplementary Figure 3

(A) Pharyngeal pumping rates of WT animals exposed to the indicated substances for one hour, shown as in Fig. 4A. ****p<0.0001, ***p<0.001, *p<0.01; ns, not significant by Bonferroni-corrected Mann-Whitney test. n = 9-86 animals.

(B) Mean speed for WT animals on indicated substances, shown as in Fig. 4C. Monosaccharides condition combines separate experiments of 0.5 M Glucose, Galactose, and Maltose. ****p<0.0001, ***p<0.001, **p<0.005, *p<0.01, by Bonferroni-corrected Mann-Whitney test. n = 15-125 animals per condition across at least three independent days.

bioRxiv preprint doi: https://doi.org/10.1101/2025.09.03.674032; this version posted September 3, 2025. The copyright holder for this preprint (which was not certified by peer review) is the author/funder, who has granted bioRxiv a license to display the preprint in perpetuity. It is made available under aCC-BY 4.0 International license.

(C) Mean GCaMP signal from NSM in *unc-13(s69)* animals on indicated conditions, shown as in Fig. 1C. ****p<0.0001 by Mann-Whitney test. n = 10-51 animals.

(D) Scatter plot showing that increased pumping does not increase NSM activity in the presence of *E. coli* + prodigiosin. Circle dots represent mean NSM GCaMP signals from *unc-13(s69)* animals under various conditions without aldicarb. Triangles represent mean NSM GCaMP signals from animals treated with 0.5 mM aldicarb to increase pumping. Arrows connect data from the same experimental conditions where the only difference is aldicarb addition. Error bars are SEM. n=8-44 animals.

bioRxiv preprint doi: https://doi.org/10.1101/2025.09.03.674032; this version posted September 3, 2025. The copyright holder for this preprint (which was not certified by peer review) is the author/funder, who has granted bioRxiv a license to display the preprint in perpetuity. It is made available under aCC-BY 4.0 International license.

Supplementary Table 1

Supplemental Table 1

Bacteria	Activity in NSM (x over No Food)	Size (µm)	Morphology	Pathogenicity	Gram-Stain	Phylum	Class	Order	Family
E. coli	2x	1 x 2-4	Rod (Bacillus)	Opportunistic	Gram-negative	Proteobacteria	Gammaproteobacteria	Enterobacterales	Enterobacteriaceae
Rhodospirillum rubrum	2.7x	0.8-1.0 x 2-5	Spiral (Spirillum)	Non-pathogenic	Gram-negative	Proteobacteria	Alphaproteobacteria	Rhodospirillales	Rhodospirillaceae
Sphingobacterium spp.	2.4x	0.5-1 x 2-5	Rod (Bacillus)	Opportunistic	Gram-negative	Bacteroidota	Sphingobacteriia	Sphingobacteriales	Sphingobacteriaceae
Stenotrophomonas spp.	2.3x	0.7-1.3 x 1.5-3	Rod (Bacillus)	Opportunistic	Gram-negative	Proteobacteria	Gammaproteobacteria	Xanthomonadales	Xanthomonadaceae
Chryseobacterium spp.	2.3x	0.5-1 x 1-4	Rod (Bacillus)	Opportunistic	Gram-negative	Bacteroidota	Flavobacteriia	Flavobacteriales	Weeksellaceae
Serratia marcescens	2x	0.5-1 x 0.9-2	Rod (Bacillus)	Opportunistic	Gram-negative	Proteobacteria	Gammaproteobacteria	Enterobacterales	Enterobacteriaceae
Pseudomonas aeruginosa	2x	0.5-0.8 x 1.5-3	Rod (Bacillus)	Opportunistic	Gram-negative	Proteobacteria	Gammaproteobacteria	Pseudomonadales	Pseudomonadaceae
Delftia	1.6x	0.5-1 x 2-5	Rod (Bacillus)	Opportunistic	Gram-negative	Proteobacteria	Betaproteobacteria	Burkholderiales	Comamonadaceae
Achromobacter spp.	1.6x	0.5-1 x 1-3	Rod (Bacillus)	Opportunistic	Gram-negative	Proteobacteria	Betaproteobacteria	Burkholderiales	Alcaligenaceae
Comamonas aquatica	1.4x	0.3-0.8 x 1.5-3	Rod (Bacillus)	Non-pathogenic	Gram-negative	Proteobacteria	Betaproteobacteria	Burkholderiales	Comamonadaceae
Staphylococcus spp.	2.5x	0.5-1	Spherical (Coccus)	Opportunistic	Gram-positive	Firmicutes	Bacilli	Bacillales	Staphylococcaceae
Leucobacter spp.	2.5x	0.5-1 x 1-3	Rod (Bacillus)	Non-pathogenic	Gram-positive	Actinobacteria	Actinobacteria	Micrococcales	Microbacteriaceae
Lactococcus spp.	2.4x	0.5-1.2	Spherical (Coccus)	Non-pathogenic	Gram-positive	Firmicutes	Bacilli	Lactobacillales	Streptococcaceae
Micrococcus luteus	2.2x	0.5-3	Spherical (Coccus)	Non-pathogenic	Gram-positive	Actinobacteria	Actinobacteria	Micrococcales	Micrococcaceae
Microbacterium nematophilum	2x	0.5-1 x 1-3	Rod (Bacillus)	Non-pathogenic	Gram-positive	Actinobacteria	Actinobacteria	Micrococcales	Microbacteriaceae
Bacillus megaterium	2x	1.5-4 x 5-20	Rod (Bacillus)	Non-pathogenic	Gram-positive	Firmicutes	Bacilli	Bacillales	Bacillaceae
Bacillus cereus	2x	1 x 3-4	Rod (Bacillus)	Pathogenic (foodborne)	Gram-positive	Firmicutes	Bacilli	Bacillales	Bacillaceae
Bacillus subtilis	2x	0.7-0.8 x 2-3	Rod (Bacillus)	Non-pathogenic	Gram-positive	Firmicutes	Bacilli	Bacillales	Bacillaceae

Table 1 shows the characteristics for all the bacteria used in this study. Activity in NSM column is the average fluorescent intensity fold increase over the negative control (i.e. No Food). Strain class column is from Samuel et al., unless otherwise referenced.
